# Spectral Fingerprint Investigation in the near Infra-Red to Distinguish Harmful Ethylene Glycol from Isopropanol in a Microchannel †

**DOI:** 10.3390/s22020459

**Published:** 2022-01-08

**Authors:** Elisabetta Bodo, Sabina Merlo, Valentina Bello

**Affiliations:** Department of Electrical, Computer and Biomedical Engineering, University of Pavia, 27100 Pavia, Italy; elisabetta.bodo01@universitadipavia.it (E.B.); valentina.bello01@universitadipavia.it (V.B.)

**Keywords:** absorption spectroscopy, ethylene glycol, glass micro-capillary, harmful alcohols, isopropanol, microfluidics, mixtures, near infra-red, optical detection, remote sensing

## Abstract

Ethylene glycol (EG) and isopropanol (ISO) are among the major toxic alcohols that pose a risk to human health. However, it is important to distinguish them, since EG is more prone to cause renal failure, and can thus be more dangerous when ingested than ISO. Analysis of alcohols such as isopropanol and ethylene glycol generally can be performed with a complex chromatographic method. Here, we present an optical method based on absorption spectroscopy, performed remotely on EG-ISO mixtures filling a microchannel. Mixtures of ethylene glycol in isopropanol at different volume concentrations were analyzed in a contactless manner in a rectangular-section glass micro-capillary provided with integrated reflectors. Fiber-coupled broadband light in the wavelength range 1.3–1.7 µm crossed the microchannel multiple times before being directed towards an optical spectrum analyzer. The induced zig-zag path increased the fluid–light interaction length and enhanced the effect of optical absorption. A sophisticated theoretical model was developed and the results of our simulations were in very good agreement with the results of the experimental spectral measurements. Moreover, from the acquired data, we retrieved a responsivity parameter, defined as power ratio at two wavelengths, that is linearly related to the EG concentration in the alcoholic mixtures.

## 1. Introduction

The ingestion of toxic alcohols including ethylene glycol (EG) and isopropanol, also known as isopropyl alcohol (ISO), is a severe health problem, since these compounds, and in particular their metabolites, may cause irreversible organ damage and central nervous system (CNR) depression. The ingestion of these alcoholic substances is treated as a medical emergency [1]. 

EG is a colorless, odorless, sweet but poisonous alcohol found in many household products. Due to the sweetness of this organic compound, used in many industrial processes, EG can be dangerous especially for children who may be exposed to it through accidental ingestion [2]. According to the 37th Annual Report of the American Association of Poison Control Centers’ National Poison Data System, there were 1070 cases of EG poisoning mentioned in 2019. Children under the age of 12 made up 177 (134 under the age of 5 and 43 between 6 and 12 years old) of the known total 1070 cases, with 22 unknown child age cases [3]. 

The major use of EG is in antifreeze and cryogenic liquids in automobiles and air-conditioning systems; it is also used as deicing fluid for windshields and aircraft, desiccant for natural gas production, precursor for manufacture of polyester fibers and resins [4,5,6]. EG is contained in the refill liquids (e-liquids) for electronic cigarettes: recent reports have revealed an increasing number of cases of accidental exposure to e-liquids, mainly through ingestion. In this context, EG may causes irreversible health damage and its detection become essential: although vapors of electronic cigarettes seem to be less toxic than tobacco smoke, there is still the need to know more about the toxicity and content of refill liquids [7]. The metabolites of EG, formed after ingestion, can be responsible for extensive cellular damage to various tissues, especially kidneys: EG undergoes biotransformation to glycolic and oxalic acids. Alcohol-related intoxication including ethylene glycol can present high anion gap metabolic acidosis and increased serum osmolar gap, whereas isopropanol intoxication happens with hyperosmolarity alone [1]. Whereas isopropanol is less harmful because it is metabolized to acetone, which is a normal component of human metabolism [8], poisoning due to EG causes disturbances in the metabolism pathways, including metabolic acidosis. Conventional treatment of this poisoning usually consists of intravenous administration of ethanol with or without hemodialysis [9]. It is thus important to differentiate isopropanol from ethylene glycol, which is more dangerous when ingested. Isopropyl alcohol does not cause renal failure like ethylene glycol. EG and ISO are miscible with many polar solvents such as water, alcohols, glycol ethers and acetone, whereas they are slightly soluble in non-polar solvents. Many efforts have been devoted to understand fundamental aspects regarding the chemical properties, in particular the toxicity, of alcohols such as EG and ISO. The analysis of these alcohols generally can be performed with a complex chromatographic method such as gas chromatography, with or without mass spectroscopy [10,11]. This method is useful to obtain quantitative data that can be considered to treat alcoholic ingestions, but they are time-consuming because they require the analysis to be performed in a remote clinical laboratory: it is a critical issue to diagnose toxic alcohol ingestions, in particular regarding ethylene glycol. In literature, sensors based on refractive index (RI) changes and surface plasmon resonance (SPR) are reported with promising results. For example, in 2019 Li et al. developed a grating coupled SPR concentration sensor to perform measurement of ethylene glycol solution concentration [12].

Another powerful optical technique is near infra-red (NIR) spectroscopy, which is nowadays a well-established method used for analytical purpose to investigate chemical compounds. Spectroscopy methods enable highly selective and sensitive detection of analytes at low concentrations. The wavelength region between 800 nm and 2500 nm is a focus for pharmaceutical and chemical analysis due to the molecular vibrations of chemical bonds associated with C–H, N–H and O–H transitions [13,14]. The absorption spectrum as a function of the wavelength is a “fingerprint” for each molecule.

Recently, capillary-based optical sensors have attracted the attention of many researchers in order to realize high-sensitivity microfluidic sensing systems combined with NIR spectroscopy. A capillary can provide a simple optical cavity in a fluidic environment that presents several unique characteristics for enhanced sensing performance; the optofluidic architectures allows for light penetration and interaction with the fluid in the capillary [15,16,17,18,19]. However, the use of miniaturized devices leads to reduced interaction length of the light with fluid, thus limiting the sensor sensitivity. To overcome this limitation, microfluidic channels are usually designed to catch the optical probe in the fluid under analysis, enabling an enhanced optical interaction with the sample and hence an increased sensitivity. However, the use of an intrusive probe limits the field of application of the sensors. The use of capillary waveguides, together with enhanced interaction length and NIR spectroscopy, offers a new possibility for optical investigation in a totally remote, contactless, and non-invasive way.

In our previous work [20], the potential of NIR spectroscopy for chemical sensing was explored by demonstrating the ability to distinguish water and pure alcohols by means of a rectangular-section glass micro-capillary. The capillary was laid onto a bulk aluminum (Al) mirror so that light crossed the channel twice before being collected and analyzed by an optical spectrum analyzer (OSA). Experimental testing was carried out by flowing deionized water, ethanol, isopropanol and ethylene glycol in the micro-capillary. We obtained a different spectral absorption profile for each tested substance in the wavelength region from 1 μm to 1.7 μm and they were in agreement with the prediction provided by the developed theoretical model.

In [21], we analyzed mixtures of ethylene glycol in isopropanol at different volume concentrations, exploiting a capillary provided with integrated reflectors, deposited by sputtering on the external surface of top and bottom glass layers: this low-cost technology allowed us to obtain a more compact sensing platform. Light crossed the channel multiple times before being directed towards the OSA, thus increasing the total optical pathlength into the channel: the effect of absorption was enhanced.

The integrated micro-fluidic platform was further explored in [22] to develop an optical sensor based on amplitude detection for specific sensing of water in ethanol–water solutions. Towards this aim, we identified a responsivity parameter obtained considering the ratio between the output power at two selected wavelengths: the 1.45 μm wavelength was explored since it corresponds to an absorption peak of water [23,24], whereas the 1.3 μm wavelength was selected as a reference since water and ethanol are only weakly absorbing.

In this work, we extended our previous investigations of EG and ISO mixtures by filling the channel with solutions of EG-ISO in different concentration. A sophisticated theoretical model based on analytical equations was developed: the theoretical results obtained inserting in the equations the values of extinction coefficients reported in the literature by other authors were found in very good agreement with the results of the experimental spectral measurements. Moreover, from the acquired data, we identified that the change in the transmitted power in the wavelength range around 1.46 µm was specifically and strongly dependent on the concentration of EG. We then retrieved a responsivity parameter defined as power ratio at two wavelengths, 1.46 µm and 1.30 µm selected as reference, that is linearly related to the EG concentration in the alcoholic mixtures. The integration of ultra-thin top and bottom Aluminum layers has allowed the fabrication of a smart devices provided with integrated reflectors, so that light can bounces multiple times (in a so called “multiple bounce configuration” [25]) inside the capillary channel to reconstruct the spectral absorption profile of the samples in more detail with respect to the single bounce configuration, in which the light crosses the microchannel only twice, simply bouncing on the bottom reflector. Enhanced EG sensitivity is achieved thanks to the multiple bounce configuration. For the first time, to the best of our knowledge, we have demonstrated the integration of rectangular glass micro-capillaries in a micro-opto-fluidic setup for detecting the spectroscopic features of EG, ISO and EG-ISO mixtures in the near infrared region. Towards this aim, we propose a miniaturized microfluidic platform based on NIR spectroscopy by using simple and low-cost instrumentation with respect to other instruments such as FTIR spectrometers, that are mature benchtop devices but very bulky and expensive, more complex in terms of design, and require a moving mirror and expensive optical instrumentation.

## 2. Materials and Methods

### 2.1. Optoelectronic Instrumental Configuration

**Figure 1:**
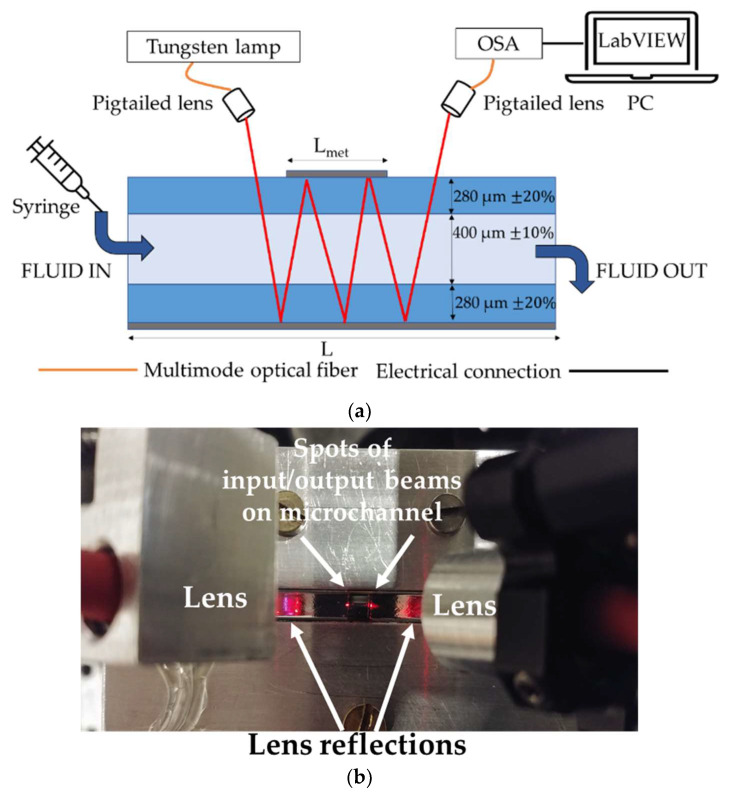
Measurement system. (**a**) Schematic representation of the optoelectronic configuration for performing absorption spectroscopy in a glass microchannel (longitudinal view). (**b**) Picture of the measurement system (top view) showing the capillary with top and bottom aluminum layers as reflectors.

The instrumental configuration for contactless, label-free and remote sensing of fluids is shown in Figure 1a. The broadband radiation provided by a tungsten lamp is fiber-coupled and shone on a rectangular glass micro-capillary using a pigtailed lens at an angle of approximately 35°. The micro-capillary investigated in this work, provided by Vitrocom (Mountain Lakes, NJ, USA), has nominal thickness of the walls of 280 μm, whereas the channel is 400 μm deep. It is particularly suitable for optical detection of fluids, since it is realized in a biocompatible material and allows the contactless remote analysis of ultra-low volumes of sample. The extremities of the capillary are inserted in heat-shrink tubing provided with luer connections: the sample fluid can be easily injected into the channel using a syringe and ejected by flowing air in the capillary. The bottom layer of the capillary is coated for its entire length *L*, equal to 5 cm, with a 50 nm-thick Al layer, while the top layer presents a 5 mm-long (*L*_met_) Al layer. These reflective layers act as integrated mirrors, inducing zig-zag light propagation through the structure. In this way, incident light crosses the fluid multiple times in a so-called multiple bounce configuration [25]. The output light is then collected by a lens, identical to the input one, and it is finally directed toward the monochromator input of an optical spectrum analyzer (OSA Agilent 86142B, Saratoga, CA, USA). A laptop is used for data collection. Signal post-processing is performed in MATLAB environment. Figure 1b shows a picture of the capillary illuminated by red light provided by a He-Ne laser: it is used in the preliminary alignment phase and for illustrative purposes.

### 2.2. The Theoretical Model

A sophisticated theoretical model was developed and implemented in MATLAB environment to predict spectroscopic analytical results in order to compare them with experimental outcomes. It was based on the geometrical ray optics approximation that is suitable to describe a light beam travelling through the micro-capillary with integrated mirrors. It can be modeled as overlaid parallel layers consisting in a channel for liquid flow, two glass barriers and thin Al layers. The model keeps in consideration the dimensions of the capillary, the real part of the refractive index (RI) of air (*n_air_* = 1), glass (*n_glass_*(*λ*)) and the complex refractive index of the sample fluid, modeled as: *n*(λ) = *n_fluid_*(*λ*) − *i*∙*k_fluid_*(*λ*), where *n_fluid_*(*λ*) and *k_fluid_*(*λ*) are the real part and the imaginary part of the refractive index, respectively; *i* is the imaginary unit.

**Figure 2:**
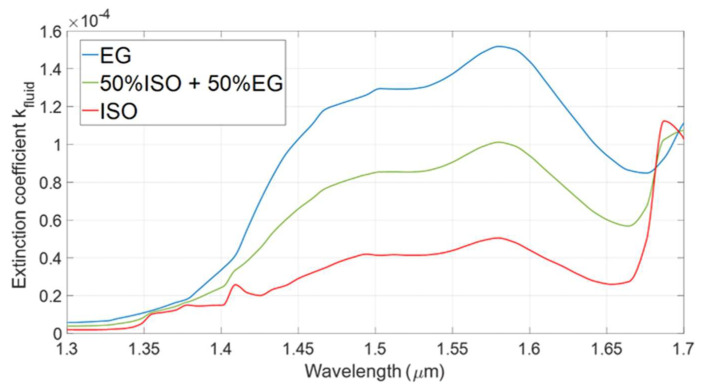
Extinction coefficient *k_fluid_*(*λ*) of isopropanol, ethylene glycol and solution of 50% of isopropanol (ISO) and 50% of ethylene glycol (EG) as functions of the wavelength. Data used to obtain this graph were found in [26,27] and they agreed with those reported in other sources [28,29,30].

In particular, the absorption features of the sample fluid depend on *k_fluid_*(*λ*) of the refractive index, also called the extinction coefficient: its wavelength behavior depends on the spectral absorption characteristics of the composing molecules, weighted by their fractional composition, and becomes a unique “fingerprint” for each molecule. Figure 2 shows the extinction coefficient (i.e., the imaginary part of the refractive index) as a function of the wavelength of pure isopropanol (‘ISO’, red trace) and pure ethylene glycol (‘EG’, blue trace): data tabulated in [26,27] are interpolated with MATLAB function *interp1* using the method *pchip*, a shape-preserving piecewise cubic interpolation. Data reported in [26,27] are in perfect agreement with those found in other scientific data collections and publications [28,29,30]. The mixing rule [31,32,33] was applied to retrieve the complex refractive index, as a function of the wavelength, of a solution composed of 50% isopropanol and 50% ethylene glycol (green trace).

**Figure 3:**
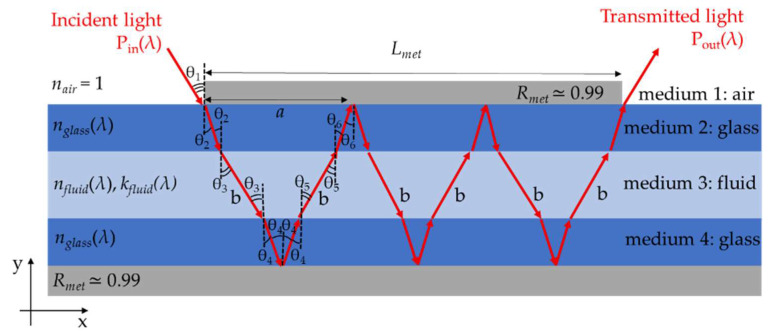
Schematic representation of the longitudinal sections of the rectangular glass capillary and light path travelled by light in case of *N* = 3. *P*_in_(*λ*) is the input optical power, *P*_out_(*λ*) is the output optical power.

Light beam propagation through the optofluidic structure is described by applying the Fresnel formulas recursively. The transmission coefficient for the s- and p- polarized fields are calculated at each interface and the mathematical average is computed to obtain the overall transmission coefficient *t*_ij_. The power transmission coefficient at each interface is calculated by applying the formula:

*T_ij_* = (*n_j_*∙*cosθ_j_*)/(*n_i_*∙*cosθ_i_*)∙|*t*_ij_|^2^ (1)

where *n_i_* and *n_j_* are the RIs (real part) of the incoming and outgoing medium, respectively, *θ_i_* is the incidence angle and *θ_j_* is the transmission angle. When light reaches the Al layers, it undergoes mirror-like reflections with a power reflectance assumed to be *R_met_* ≈ 0.99. The total optical path *B* travelled by the radiation into the fluidic channel depends on the number of bounces *N* and it is given by:

*B* = 2 ∙ *b* ∙ *N* (2)

where *b* is the distance travelled by light each time it crosses the channel containing the sample fluid, as shown in Figure 3. It is calculated as follows:

*b* = *d*/*cosθ*_3_ (3)

*N* is estimated by computing:

*N* = *CEIL* [*L_met_*/*a*] (4)

where *a* is the distance travelled by light along the x-direction at each bounce; CEIL is the Matlab function that returns the smallest integer value that is larger than, or equal to, *L_met_/a*. Figure 3 shows the light-path travelled by light in the case of a number of bounces *N* = 3.

By applying the Lambert–Beer formula, the transmittance due to the fluid absorbance as a function of the wavelength can be calculated as:

*T_abs_*(*λ*) = *e*^−*α*(*λ*) ∙ *B*^ (5)

where *α(λ)* is the absorption coefficient that depends on *k_fluid_*(*λ*):

*α*(*λ*) = 4 ∙ *π* ∙ *k_fluid_*(*λ*)/*λ* (6)

Eventually, the spectral behavior of the output light 𝑃_𝑜𝑢𝑡_(𝜆) can be found by multiplying all the contributions of transmittance. In the presence of an absorbing fluid sample in the capillary channel, the spectral transmittance is given by:

*T_sample_*(*λ*) = *P_out_*(*λ*)/*P_in_*(*λ*) = *T*_12_ ∙ *T*_23_*^N^* ∙ *T*_34_*^N^* ∙ *T*_43_*^N^* ∙ *T*_32_*^N^* ∙ *T*_21_ ∙ *T_abs_*(*λ*) ∙ *R_met_*^2∙*N*−1^ (7)

where *P_in_*(*λ*) is the input optical power and *P_out_*(*λ*) is the output optical power*. P_in_*(*λ*) is considered constant over the wavelength range of interest.

## 3. Results and Discussion

### 3.1. Spectral Analysis

**Figure 4:**
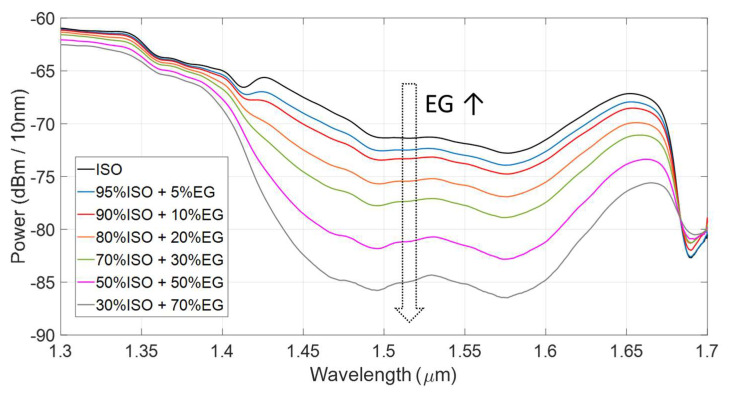
Experimental power spectra acquired when filling the capillary channel with isopropanol and isopropanol-ethylene glycol mixtures in various concentrations.

Experimental measurements were carried out by filling the channel with mixtures of EG and ISO in different concentrations. Ethylene glycol anhydrous, 99.8% pure, was purchased by Sigma-Aldrich (St. Louis, MO, USA). Isopropanol (RS Pro Isopropyl Alcohol Cleaner) was purchased from RS Components (London, UK). Figure 4 shows the transmitted power spectra collected by testing pure isopropanol (black trace) and solutions of ethylene glycol in isopropanol in volume concentrations equal to 5% (blue trace), 10% (red trace), 20% (orange trace), 30% (green trace), 50% (pink trace) and 70% (grey trace). For increasing EG concentration, the transmitted power between 1.40 μm and 1.60 μm decreases since ethylene glycol exhibits two absorption bands around 1.46 μm and 1.57 μm, as illustrated in Figure 2.

**Figure 5:**
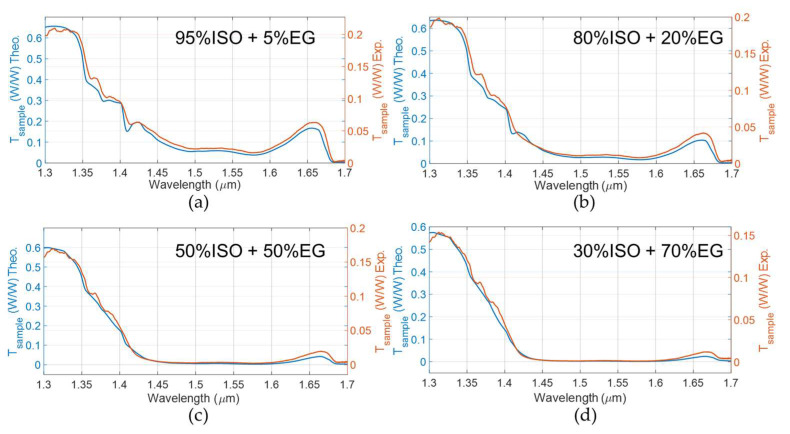
Theoretical (‘Theo.’, blue trace) and experimental (‘Exp.’, orange trace) T*_sample_*(λ) for solutions of ethylene glycol in isopropanol: (**a**) solution with EG concentration *C* = 5%; (**b**) solution with EG concentration *C* = 20%; (**c**) solution with EG concentration *C* = 50%; (**d**) solution with EG concentration *C* = 70%. All the theoretical traces of *T_sample_(λ)* were obtained by applying our developed model using values of extinction coefficients for EG and ISO reported in [26,27].

The theoretical transmittance *T_sample_*(*λ*) (Equation (7)) was calculated for performing a comparison with the line-shape of the experimental spectral data. Figure 5 shows the results obtained when considering solutions of EG in ISO in volume concentrations *C* of EG equal to 5%, 20%, 50% and 70%, in the wavelength range from 1.3 µm to 1.7 μm. The blue trace is the theoretical *T_sample_*(*λ*) obtained by applying the developed model using values of extinction coefficients for EG and ISO reported in [26,27]: a number of bounces equal to *N =* 7 was found for the top mirror length considered. The orange trace represents the experimentally detected transmittance. The ray tracing model neglects additional losses due to non-ideal guiding properties of the capillary: the guided propagation of the light beam, which is due to the presence of the aluminum, occurs only in the vertical plane. Moreover, the model neglects losses that are not due to fluid absorption or to the partial transmission at the interfaces of the micro-fluidic platform: in the actual condition, coupling losses between inlet and outlet, which are almost constant with the wavelength, are neglected.

**Figure 6:**
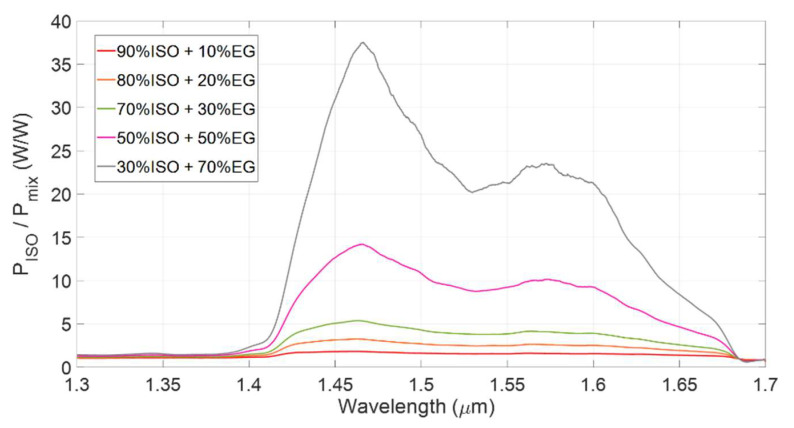
Spectral ratio between the transmitted power collected by flowing into the channel mixtures of isopropanol with ethylene glycol, in different concentrations.

To exploit this method for quantifying EG content in the solution, the ratio between the transmitted power obtained for pure isopropanol (*P_ISO_*), thus playing the role of reference fluid of this experiment, and the transmitted power obtained when testing the mixtures (*P_mix_*) was considered. Figure 6 shows the experimental spectral results of the ratio *P_ISO_*/*P_mix_* for different volume concentrations *C* of ethylene glycol in isopropanol: red trace *C* = 10%, orange trace *C* = 20%, green trace *C* = 30%, pink trace *C* = 50% and grey trace *C* = 70%. The ratio *P_ISO_/P_mix_* was explored to study the dependence on EG concentration of the transmitted power spectra. In particular, the narrow wavelength range around *λ* = 1.46 μm shows the highest rate of change of the transmitted power as a function of the EG concentration (the derivative d(*P_ISO_/P_mix_*)/d*C* reaches its maximum value), thus optical absorption of the solution is strongly dependent on ethylene glycol content. On the other hand, at *λ* = 1.35 μm both isopropanol and ethylene glycol are only weakly absorbing, and this wavelength can be taken as a reference.

### 3.2. Responsivity and Sensitivity

In view of the design of an optical sensor based on amplitude detection, the ratio of the detected power at two different wavelengths was computed. We defined the responsivity R_1.46/1.35_ as the logarithmic value (base 10) of the ratio between the output power at 1.46 μm (P_out@1.46µm_) and 1.35 μm (P_out@1.35µm_):

R_1.46/1.35_ = Log(P_out@1.46µm_/P_out@1.35µm_) (8)

**Figure 7:**
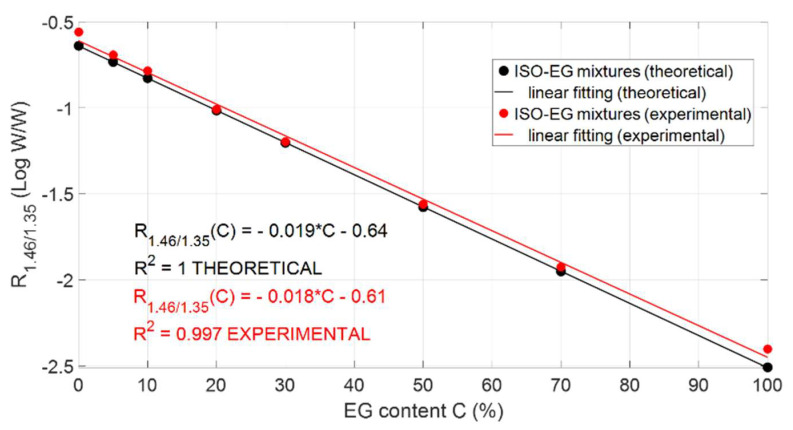
Experimental and theoretical responsivity data points (red and black dots, respectively) with best linear fittings.

By calculating the responsivity R_1.46/1.35_ for all tested solutions as a function of the ethylene glycol content *C* (%) and by linearly fitting the data, both theoretical and experimental calibration curves were retrieved. The theoretical calibration curve was calculated by considering a number of bounces equal to *N* = 7 for all tested solutions. As shown in Figure 7, they are in very good agreement: the red dots represent the experimental values *R*_1.46/1.35_ whereas the black dots are the theoretically calculated values. The red and black lines represent the best linear fittings of the experimental and theoretical data, respectively. The slope of the curves represents the sensitivity of the proposed system for detecting ethylene glycol concentration in isopropanol solutions.

## 4. Conclusions

In conclusion, the functionality of a micro-opto-fluidic platform based on rectangular glass micro-capillary to detect ethylene glycol in solutions of isopropanol by exploiting NIR absorption spectroscopy was demonstrated. The ingestion of toxic alcohols including EG and ISO is a severe health problem: it is important to distinguish EG from ISO, since EG is more prone to cause renal failure, and can thus be more dangerous than ISO when ingested. Our micro-fluidic configuration allows to investigate mixtures of fluids in a contactless manner. The deposition of thin Al layers is a simple and low-cost technology that allows the fabrication of devices, having stretched interaction length with the chemical sample and enhanced spectroscopic sensitivity, suitable for several application fields. The experimental results are in good agreement with the prediction provided by the developed model: this paves the way to the exploitation of this method, based on the preliminary modeling of the spectral properties of complex mixtures, for selecting the adequate pathlength (thus, the number of bounces) for a required sensitivity and best wavelength combination for a responsivity parameter. The sensing platform based on amplitude detection on narrow spectral bands is versatile and smart: it can be exploited to detect several solutions and substances in a specific way, simply modifying the wavelengths for the calculation of the responsivity parameter, on the basis of the absorption properties of the substance to be detected.

In the future, advanced processing techniques based on machine learning and principal component analyses will be investigated to gain more insight into the specific features of the spectral information that is more powerful for distinguishing fluid compositions. In view of industrial real-life practical applications, we plan to assemble a more compact setup using LEDs with emission spectra centered at 1.35 µm and 1.46 µm, instead of the lamp, as readout sources. Since LEDs can be driven with pulsed out-of-phase current signals, a single InGaAs photodiode can be exploited to detect instantaneously the transmitted optical power in both wavelength ranges, to easily obtain the responsivity ratio. Moreover, since the proposed sensing method is non-invasive and employs low power, future work will be devoted to the analysis of biological fluids in the biomedical and biochemical fields. Since the micro-capillaries are available not only in borosilicate glass but also in synthetic fused silica, they could be used for several IR applications in the wavelength range up to 3.6 μm. The multiple bounces configuration opens up a new way to perform absorption spectroscopy analyses on compounds with specific fingerprints in the spectral region above 1.7 µm such as uric acid, ammonia, creatinine and cholesterol and even hormones such as melatonin.

## Data Availability

Not applicable.

## References

[1] Kraut J.A., Kurtz I. (2008). Toxic Alcohol Ingestions: Clinical Features, Diagnosis, and Management. Clin. J. Am. Soc. Nephrol..

[2] Iqbal A., Glagola J.J., Nappe T.M. (2020). Ethylene Glycol Toxicity. StatPearls.

[3] Gummin D.D., Mowry J.B., Beuhler M.C., Spyker D.A., Brooks D.E., Dibert K.W., Rivers L.J., Pham N.P.T., Ryan M.L. (2020). 2019 Annual Report of the American Association of Poison Control Centers’ National Poison Data System (NPDS): 37th Annual Report. Clin. Toxicol..

[4] Yue H., Zhao Y., Ma X., Gong J. (2012). Ethylene Glycol: Properties, Synthesis, and Applications. Chem. Soc. Rev..

[5] Patočka J., Hon Z. (2010). Ethylene Glycol, Hazardous Substance in the Household. Acta Med..

[6] An L., Chen R. (2016). Recent Progress in Alkaline Direct Ethylene Glycol Fuel Cells for Sustainable Energy Production. J. Power Sources.

[7] Varlet V., Farsalinos K., Augsburger M., Thomas A., Etter J.-F. (2015). Toxicity Assessment of Refill Liquids for Electronic Cigarettes. Int. J. Environ. Res. Public Health.

[8] Greene H.R., Krasowski M.D. (2020). Correlation of Osmolal Gap with Measured Concentrations of Acetone, Ethylene Glycol, Isopropanol, Methanol, and Propylene Glycol in Patients at an Academic Medical Center. Toxicol. Rep..

[9] Lister D., Tierney M., Dickinson G. (2005). Effectiveness of IV Ethanol Therapy Combined with Hemodialysis in the Treatment of Methanol and Ethylene Glycol Poisoning. Can. J. Hosp. Pharm..

[10] McQuade D.J., Dargan P.I., Wood D.M. (2014). Challenges in the Diagnosis of Ethylene Glycol Poisoning. Ann. Clin. Biochem..

[11] Porter W.H., Rutter P.W., Yao H.H. (1999). Simultaneous Determination of Ethylene Glycol and Glycolic Acid in Serum by Gas Chromatography Mass Spectrometry. J. Anal. Toxicol..

[12] Li W., Li Z., He J., Chu L. (2019). Design and Performance of a Composite Grating-Coupled Surface Plasmon Resonance Trace Liquid Concentration Sensor. Sensors.

[13] Beć K.B., Grabska J., Huck C.W. (2021). Principles and Applications of Miniaturized Near-Infrared (NIR) Spectrometers. Chemistry.

[14] Caccamo M.T., Magazù S. (2017). Ethylene Glycol–Polyethylene Glycol (EG-PEG) Mixtures: Infrared Spectra Wavelet Cross-Correlation Analysis. Appl. Spectrosc..

[15] Chen S., Liu Y., Yu Q., Peng W. (2020). A Novel Visible Light-Excited Interference Behavior Occurred in Capillary Waveguide. IEEE Sens. J..

[16] Salim A., Lim S. (2018). Review of Recent Metamaterial Microfluidic Sensors. Sensors.

[17] Borecki M., Korwin-Pawlowski M.L., Beblowska M., Szmidt J., Jakubowski A. (2010). Optoelectronic Capillary Sensors in Microfluidic and Point-of-Care Instrumentation. Sensors.

[18] Alberti S., Datta A., Jágerská J. (2021). Integrated Nanophotonic Waveguide-Based Devices for IR and Raman Gas Spectroscopy. Sensors.

[19] Wang C., Sahay P. (2009). Breath Analysis Using Laser Spectroscopic Techniques: Breath Biomarkers, Spectral Fingerprints, and Detection Limits. Sensors.

[20] Bello V., Bodo E. (2020). A NIR-Spectroscopy-Based Approach for Detection of Fluids in Rectangular Glass Micro-Capillaries. Eng. Proc..

[21] Bodo E., Bello V. Microfluidic Devices with Selectable Optical Pathlength for Quality Control of Alcoholic Solutions. Proceedings of the 8th International Electronic Conference on Sensors and Application.

[22] Bello V., Bodo E., Merlo S. (2021). Micro-Opto-Fluidic Platform for Spectroscopic Identification of Water-Based Fluids. Optical Sensors 2021, Proceedings of the SPIE Optics + Optoelectronics, Online, 19–23 April 2021.

[23] Kedenburg S., Vieweg M., Gissibl T., Giessen H. (2012). Linear Refractive Index and Absorption Measurement of Nonlinear Optical Liquids in the Visible and Near-Infrared Spectral Region. Opt. Mater. Express.

[24] Curcio J., Petty C. (1951). The Near Infrared Absorption Spectrum of Liquid Water. J. Opt. Soc. Am..

[25] Bello V., Bodo E., Merlo S. Quality Control of Ethanol-Based Hand Sanitizer Gels in Micro-Opto-Fluidic Devices. Proceedings of the CLEO: Applications and Technology 2021.

[26] Sani E., Dell’Oro A. (2014). Optical Constants of Ethylene Glycol Over an Extremely Wide Spectral Range. Opt. Mater..

[27] Sani E., Dell’Oro A. (2016). Spectral Optical Constants of Ethanol and Isopropanol from Ultraviolet to Far Infrared. Opt. Mater..

[28] John Wiley & Sons, Inc. SpectraBase; SpectraBase Compound ID=8Ro8UyUNHr6 SpectraBase Spectrum ID=5rDQoxoqFzV. https://spectrabase.com/spectrum/5rDQoxoqFzV.

[29] John Wiley & Sons, Inc. SpectraBase; SpectraBase Compound ID=9cBA9IZSWuJ SpectraBase Spectrum ID=LHQElUGVPJq. https://spectrabase.com/spectrum/LHQElUGVPJq.

[30] Myers T.L., Tonkyn R.G., Danby T.O., Taubman M.S., Bernacki B.E., Birnbaum J.C., Sharpe S.W., Johnson T.J. (2018). Accurate Measurement of the Optical Constants n and k for a Series of 57 Inorganic and Organic Liquids for Optical Modeling and Detection. Appl. Spectrosc..

[31] Abo Riziq A., Erlick C., Dinar E., Rudich Y. (2007). Optical Properties of Absorbing and Non-Absorbing Aerosols Retrieved by Cavity Ring Down (CRD) Spectroscopy. Atmos. Chem. Phys..

[32] Flores J.M., Trainic M., Borrmann S., Rudich Y. (2009). Effective Broadband Refractive Index Retrieval by a White Light Optical Particle Counter. Phys. Chem. Chem. Phys..

[33] Bain A., Rafferty A., Preston T.C. (2019). The Wavelength-Dependent Complex Refractive Index of Hygroscopic Aerosol Particles and Other Aqueous Media: An Effective Oscillator Model. Geophys. Res. Lett..

